# Genetic and Technological Characterisation of Vineyard- and Winery-Associated Lactic Acid Bacteria

**DOI:** 10.1155/2015/508254

**Published:** 2015-03-19

**Authors:** Aspasia A. Nisiotou, Dimitra Dourou, Maria-Evangelia Filippousi, Ellie Diamantea, Petros Fragkoulis, Chryssoula Tassou, Georgios Banilas

**Affiliations:** ^1^Wine Institute of Athens, ELGO “DEMETER”, S. Venizelou 1, 15341 Lykovrysi, Greece; ^2^Department of Enology, Technological Educational Institute of Athens, Ag. Spyridonos Street, 12210 Aegaleo, Greece; ^3^Institute of Technology of Agricultural Products, ELGO “DEMETER”, S. Venizelou 1, 15341 Lykovrysi, Greece

## Abstract

Vineyard- and winery-associated lactic acid bacteria (LAB) from two major PDO regions in Greece, Peza and Nemea, were surveyed. LAB were isolated from grapes, fermenting musts, and winery tanks performing spontaneous malolactic fermentations (MLF). Higher population density and species richness were detected in Nemea than in Peza vineyards and on grapes than in fermenting musts. *Pediococcus pentosaceus* and *Lactobacillus graminis* were the most abundant LAB on grapes, while *Lactobacillus plantarum* dominated in fermenting musts from both regions. No particular structure of *Lactobacillus plantarum* populations according to the region of origin was observed, and strain distribution seems random. LAB species diversity in winery tanks differed significantly from that in vineyard samples, consisting principally of *Oenococcus oeni*. Different strains were analysed as per their enological characteristics and the ability to produce biogenic amines (BAs). Winery-associated species showed higher resistance to low pH, ethanol, SO_2_, and CuSO_4_ than vineyard-associated isolates. The frequency of BA-producing strains was relatively low but not negligible, considering that certain winery-associated *Lactobacillus hilgardii* strains were able to produce BAs. Present results show the necessity of controlling the MLF by selected starters in order to avoid BA accumulation in wine.

## 1. Introduction

In winemaking, a secondary fermentation known as malolactic fermentation (MLF) often takes place following the cease of yeast activity. During MLF, L-malate is converted into L-lactate by the lactic acid bacteria (LAB) of wine. This bioconversion is a desirable process in red winemaking and also in the production of certain white wines of high acidity, due to the organoleptic advantages that LAB activity confers. These include a decline in the total acidity and an increase of soft mouth feel, flavour, and microbiological stability of the wine [1]. However, MLF often entails certain risks, that is, the production of off-flavours, reduction in colour, and most importantly the formation of biogenic amines (BAs) [2, 3].

Currently, there is a growing concern regarding the limits of BAs in wines because of their potential health implications [4]. Although not regulated uniformly worldwide, BAs are generally confronted under similar regulations as for allergens. As a matter of fact, wines containing elevated amounts of histamine are rejected from certain markets due to recommended or suggested existing limits [4], while recently the Panel on Biological Hazards of the European Food Safety Authority (EFSA) released a scientific opinion on risk based control of BA formation in fermented foods [5]. Therefore, MLF in wine needs to be regulated to avoid the accumulation of BAs by LAB. This may be accomplished by the use of selected LAB strains tested for low production of BAs [6, 7] or able to degrade BA in wine [8].

Selected strains of* Oenococcus oeni*, the principal malolactic bacterium, have been launched in the market over the last decades. Nevertheless, wineries often face difficulties when conducting MLF by current commercial starters, as the induction of the process is not always successful [9]. Still several wineries prefer to conduct spontaneous malolactic fermentations by the native microbiota [10]. In these cases, the indigenous bacteria actualize MLF more effectively than commercial* O. oeni*, since native strains can deal with microbial incompatibilities and are better acclimatized to the local wine and practices [11, 12]. In addition, spontaneous MLF typically involves a composite bacterial community that may confer a more complex flavour to wine [1].

To this end, the wine industry seeks for novel MLF starters bearing positive technological and flavouring attributes [12]. The use of LAB species other than* O. oeni* is also being considered [13]. Grape resident microbial diversity forms an untapped reservoir of indigenous bacteria strains and may be primarily considered in an MLF starter selection scheme. Here we explored the local vineyard- and winery-associated LAB culturable populations in two key viticultural regions in Greece, Nemea and Peza. By using different molecular techniques various species and strains of enological importance were identified and characterised.

## 2. Materials and Methods

### 2.1. Sampling and LAB Isolation

Grape samples belonging to the Greek grapevine (*Vitis vinifera*) varieties “Vilana” (white), “Mandilaria” (red), and “Kotsifali” (red) were collected from 16 vineyards (1VP–16VP) within the Peza PDO region in Crete. Grapes of the “Agiorgitiko” cultivar (red variety) were collected from 11 vineyards (1VN–11VN) in the Nemea PDO region, Peloponnese. Samples consisting of healthy grape bunches were collected from at least 3 distant sampling points (sites) within each vineyard, placed into sterile plastic bags and transferred at 4°C to the laboratory. Grapes were crushed with a stomacher and let to ferment spontaneously in sterile bottles. Fermentation progress was daily followed by weight determinations. LAB were isolated from grapes or fermenting grape juice at the middle stage (MF) when about 50% of sugars were consumed, the final stage (EF) when sugars were depleted, and after the end of alcoholic fermentation. LAB were also isolated from wine samples collected from 9 tanks (T1–T9) of a winery in Nemea during spontaneous MLF. No spontaneous MLF was conducted in Peza winery. For bacteria enumeration, appropriate dilutions were spread onto MRS agar medium (pH 5.5) supplemented with 100 mg/L cycloheximide and incubated in anaerobic jars at 28°C for 3–8 days. Colonies were randomly selected from plates and examined microscopically. Bacterial colonies were further examined for Gram stain and catalase reaction. Isolates were maintained in liquid cultures in MRS broth with 30% glycerol at −80°C until further analysis.

### 2.2. Species Identification

DNA was extracted as previously described [14]. The 16S rDNA region of bacteria isolates was PCR-amplified using primers pA and pH [15]. For restriction analysis of the amplified 16S rDNA region (16S-ARDRA), approximately 500 ng of PCR product was digested with the restriction endonuclease* Mse*I [15] and fragments were analyzed by agarose gel electrophoresis. For the differentiation of* Lactobacillus plantarum*,* Lactobacillus pentosus*, and* Lactobacillus paraplantarum*, a multiplex PCR assay was performed with the* recA* gene-based primers paraF, pentF, planF, and pREV, according to Torriani et al. [16]. For sequence analysis, the V1–V3 region of 16S rDNA was amplified using the primers P1V1 and P4V3 as previously described [17]. PCR products of representative isolates per distinct PCR-ARDRA pattern were sequenced (Macrogen; http://www.macrogen.com/). BLAST searches of sequences were performed at the NCBI/GenBank database.

### 2.3. Strain Typing and Genetic Analysis

Repetitive element sequence-based PCR (rep-PCR) using the single primer (GTG)5 or the primer pair REP1R-Dt and REP2R-Dt [18, 19] and RAPD analysis using the single primer RAPD1 or RAPD2 [20], 5′-ACGCGCCCT-3′ [21], and 1283 [22] were initially evaluated. The banding patterns corresponding to isolates from the same vineyard were considered as a vineyard population. UPGMA clustering of vineyard populations was conducted by using the PopGene 1.32 software [23].

### 2.4. Detection of BA-Producing Genes

For simultaneous detection of four genes involved in the production of major BAs in wine by LAB, that is, histamine (*hdc*), tyramine (*tyrdc*), and putrescine (*odc* and* agdi*), a multiplex PCR assay was applied as described elsewhere [24]. Briefly, the* hdc* and* tyrdc* genes were targeted with the primer pairs HDC3/HDC4 and TD2/TD5, respectively, while the primers ODC1/ODC2 and AGD1/AGD2 were used for the detection of* agdi* and* odc* genes, respectively. The 16S rRNA gene was concomitantly targeted with the universal primers BSF8/BSR1541 [25].

### 2.5. Technological Characterization of LAB

Tests were performed on MRS agar (pH 4) containing 7% ethanol unless otherwise stated. Ethanol tolerance was determined at ethanol contents of 10, 12, or 14%. SO_2_ resistance was evaluated at 5, 15, or 30 mg/L. Tolerance to low pH was determined at pH values of 3.0, 3.5, 4.0, or 5.5 adjusted by the addition of HCl. CuSO_4_ tolerance was evaluated at concentrations of 5 or 20 mg/L. Biogenic amines formation was determined on modified decarboxylating agar (MDA) plates (per litre: 5.0 g tryptone, 8.0 g meat extract, 4.0 g yeast extract, 0.5 g Tween 80, 0.2 g MgSO_4_, 0.05 g MnSO_4_, 0.04 g FeSO_4_, 0.1 g CaCO_3_, 0.06 g bromocresol purple, and 20.0 g agar) supplemented with 2% of either tyrosine, histidine, or arginine. The formation of biogenic amines was indicated by a purple halo around the bacterial colony as a result of amino acid decarboxylation [26]. Isolates were spot inoculated (ca. 10^6^ cells/mL) on the surface of agar medium. Growth was evaluated after anaerobic incubation for up to 8 days at 28°C.

## 3. Results and Discussion

### 3.1. Bacterial Abundance

Grapes and wine fermentations constitute complex microbial ecosystems consisting of highly dynamic yeast and bacteria communities. Despite the importance of LAB populations in shaping the wine quality, our current knowledge on the spatiotemporal distribution of LAB populations in grapes and musts during the alcoholic or malolactic fermentation is still limited. Here we analyzed the LAB culturable communities in two distant viticultural zones in Greece, Peza in Crete and Nemea in Peloponnese. Samples included grapes and the respective fermenting musts. Sampling was also conducted after the end of the alcoholic fermentation (AF) and* in situ* in winery tanks during spontaneous MLF.

LAB were detected at relatively low frequencies on grapes. About 28% of grape samples from the Nemea region harbored bacteria at populations ranging from 1.4 to 3.8 log CFU/mL. In grapes from Peza, the bacterial populations were below the detection limit. The low incidence of LAB populations on wine grapes, as detected here, is in accordance with previous studies that suggest limited LAB population density (<3 log CFU/g) in vineyards, due to their nutritional requirements [10, 27–31].

Musts from grape samples were allowed to ferment spontaneously and at the middle stage of the AF (MF stage) bacteria could be recovered from 16% of the samples from either region. In the case of Nemea, population densities were relatively low (1.4–3.7 log CFU/mL), except for a single population that reached 8.7 log CFU/mL. Similarly, in Peza samples, populations at stage MF ranged from 0.9 to 3.3 log CFU/mL, except for one sample (ca. 7.2 log CFU/mL). At the end of the AF (EF stage), the number of Nemea samples with detectable populations decreased to 9%, while counts ranged from 1.3 to 7.0 log CFU/mL. As opposed, the respective percentage of Peza samples increased (24%), with populations ranging from 1.9 to 4.3 log CFU/mL. No bacterial populations were detected in samples from Nemea or Peza regions after the completion of AF. Present results show that, with a few exceptions, the bacterial growth is limited during the AF. Similarly low bacterial densities during the AF, ranging from 2 to 4 log CFU/mL, have been recorded previously [32]. These populations may further decline at the end of AF, with the exception of* O. oeni* [28, 32–36]. It is most likely that bacterial growth is prevented by the accumulating ethanol, the lack of nutrients, or the competition with indigenous yeast biota [28, 36]. Contracting this general observation, tumultuous bacterial growth during AF, as reported here, has been occasionally associated with musts infected with certain* Lactobacillus* spp. [28]. As opposed to vineyard-associated samples, relatively high bacterial densities (ca. 7 log CFU/mL) were recovered from winery tanks T1–T6. Populations of ca. 4 log CFU/mL were detected in tanks T7 and T8. Bacteria were below the detection limit in tank T9.

### 3.2. Species Identification

**Figure 1:**
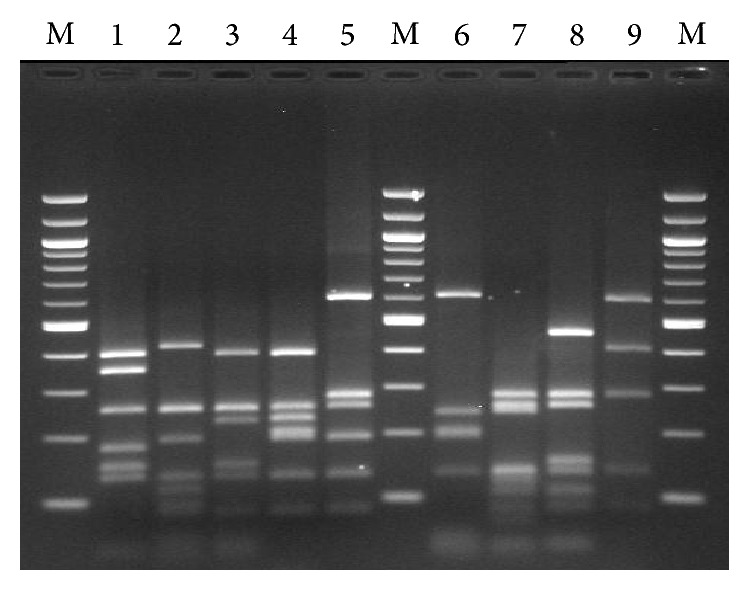
16S-ARDRA patterns obtained after digestion with* Mse*I. Lanes: 1,* Lactococcus lactis*; 2,* Lactobacillus hilgardii*; 3,* Pediococcus parvulus*; 4,* Weissella* sp.; 5,* Lactobacillus graminis*; 6,* Oenococcus oeni*; 7,* Pediococcus pentosaceus*; 8,* Lactobacillus plantarum*; 9,* Staphylococcus epidermidis*; M, 100 bp molecular marker.

16S-ARDRA grouped 626 isolates according to their banding profiles (profiles I to IX) (Table 1; Figure 1). Phylogenetic analysis of the V1–V3 region of 16S rDNA of representative isolates from each group assigned them to the species* Lactobacillus graminis*,* Lactobacillus hilgardii*,* Lactobacillus pentosus/plantarum*,* Lactococcus lactis*,* Oenococcus oeni*,* Pediococcus parvulus*,* P. pentosaceus*,* Staphylococcus epidermidis*, and* Weissella* sp. According to the above analysis, isolates within group III showed 100% sequence similarity to both* Lactobacillus pentosus* JCM 1558^T^ (D79211) and* Lactobacillus plantarum* NRRL B-14768^T^ (AJ965482) followed by 99.8% to* Lactobacillus paraplantarum* DSM 10667^T^. Since 16S rDNA sequence is identical or highly similar among these species, a multiplex PCR assay with* recA* gene-based primers was applied for the identification of isolates within group III, as previously suggested [16], revealing that all isolates belong to the species* Lactobacillus plantarum*.

### 3.3. LAB Species Diversity and Succession

*Pediococcus pentosaceus* and* Lactobacillus graminis* were the most abundant LAB species in grape samples from Nemea (12.5 and 9.4%, resp.), followed by* Weissella* sp. and* Lactococcus lactis* at percentages lower than 7%. Typically, LAB species diversity associated with grape surfaces is rather limited mainly due to their nutritional requirements [28]. Species that have been reported to occur on grapes belong to the genera* Lactobacillus* (*Lactobacillus casei*,* Lactobacillus hilgardii*,* Lactobacillus kunkeei*,* Lactobacillus lindneri*,* Lactobacillus mali*, and* Lactobacillus plantarum*),* Pediococcus*, and* Leuconostoc* [29, 37, 38]. By applying a culture independent approach Renouf et al. [39] revealed a broader LAB diversity than previously described, including species within the genera* Enterococcus* and* Weissella*. Here we also detected* Lactococcus lactis*, a species that is quite scarce on grapes and a potentially novel* Weissella* species.

At the MF stage in Nemea samples,* Pediococcus pentosaceus* showed a higher level of persistence compared to the other species encountered on grapes. All other grape-associated populations were undetectable except for* Lactobacillus graminis*, which replaced* S. epidermidis* in one case.* Lactobacillus plantarum* emerged for the first time in two out of five samples, in which initial LAB populations on grapes were below the detection limit. At the EF stage, LAB were detected in three samples and all isolates were identified as* Lactobacillus plantarum*. Although in Peza grape samples bacteria were below the detection limit, LAB populations then emerged during the AF. At stage MF,* Lactobacillus plantarum* was the only species detected in all samples. At the EF stage, all samples were exclusively dominated by* Lactobacillus plantarum*, except for one sample in which* P. pentosaceus* thrived.

Previous studies have also shown that* Lactobacillus plantarum* is scarce on grapes [29, 30], but frequent in fermenting musts [10].* Oenococcus oeni*, the principal malolactic bacterium often isolated from wines, was not detected on grapes or fermenting musts, collaborating previous suggestions about the absence or low population of this species in Greek vineyards [10].

The dominant population in winery-associated samples was* O. oeni* that could be recovered from all tanks performing spontaneous MLF. In 75% of the samples,* Pediococcus parvulus* was also isolated, albeit at significant lower populations than* O. oeni*. In one case,* Lactobacillus hilgardii* was also isolated along with* P. parvulus*, again at much lower population density than* O. oeni* (ca. 3 versus 7 log CFU/mL, resp.). The high occurrence of* P. parvulus* in the present samples needs further consideration since it is often associated with ropiness and oiliness of wine [40]. Furthermore,* P. parvulus* and* Lactobacillus hilgardii* were identified as the main spoilage, high histamine producing bacteria [41]; therefore their presence during MLF needs to be controlled.

### 3.4. Genotypic Diversity

For the discrimination of different LAB genotypes, various PCR-based fingerprinting methods were initially evaluated, including rep-PCR using the primer (GTG)5 or the primer set REP1R-Dt/REP2R-Dt and RAPD analysis with various primers. Among them, PCR using the primer RAPD2 (RAPD2-PCR) generated clear and reproducible banding patterns and also showed the highest discriminatory capacity in our tests (data not shown). Therefore, it was retained as the fingerprinting method of choice in the present genotyping analysis. The primer RAPD2 has been successfully applied previously in RAPD-PCR assays to differentiate strains within various LAB species [20, 42].

**Figure 2:**
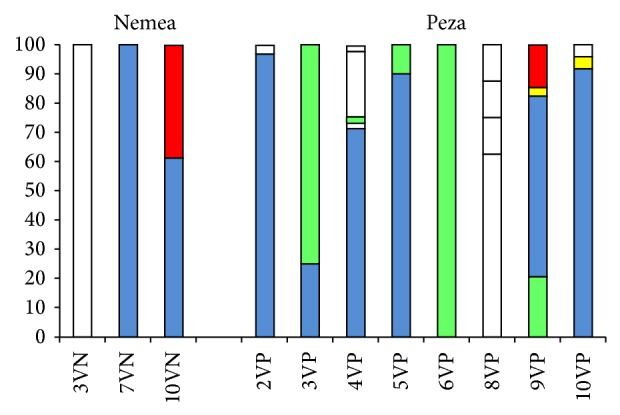
Distribution of* Lactobacillus plantarum* genotypes (%) in different vineyards of Nemea and Peza regions. Common genotypes are represented with the same colour. Unique genotypes are shown in white colour.

**Figure 3:**
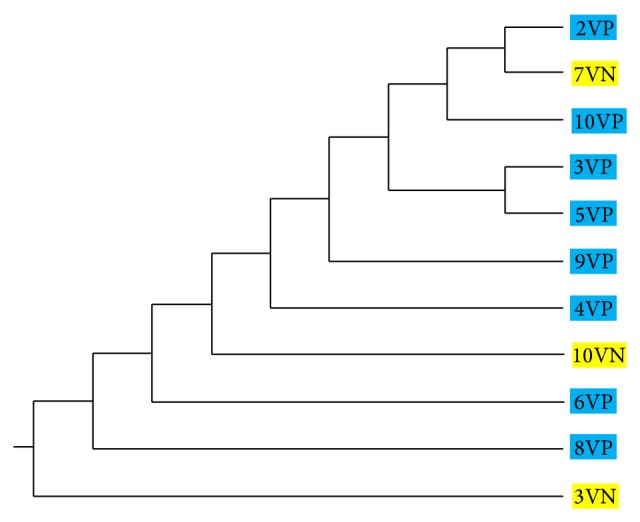
UPGMA dendrogram based on Nei's genetic distances among* Lactobacillus plantarum* vineyard populations. Populations from Nemea and Peza are yellow- and blue-highlighted, respectively.

In the case of* Lactobacillus plantarum* isolates, RAPD2-PCR generated a total of 45 polymorphic bands and 14 distinct banding patterns (hereafter referred to as genotypes) were identified (Table 2). The number of different genotypes detected within a vineyard (all sampling points included) ranged from 1 to 5 (Figure 2). Recent metagenomic studies by using next generation sequencing technology suggest that different wine-growing regions may maintain different microbial communities [43, 44]. As far as regional variation in wine characteristics may be influenced by the local grape microflora, the so-called microbial “*terroir*” concept, it is very important to examine in more detail the spatiotemporal distribution of various strains. In this study, population genetic analysis was conducted in isolates of different vineyards (populations) and the existence of genetic structure between populations of the two geographical zones of origin (groups of Peza and Nemea) was evaluated. Results from UPGMA cluster analysis showed that the spatial distribution of genotypes within a vineyard is rather random (data not shown). Measures of genetic identity (Nei's coefficient) showed that most vineyard populations shared a relatively high degree of genetic similarity (>0.7). The UPGMA tree of vineyard populations showed no clustering according to the zone of origin (Figure 3).

The isolates from four more vineyard-associated LAB populations belonging to the species* Lactobacillus graminis*,* Lactococcus lactis*,* P. pentosaceus*, and* Weissella* sp. were analysed by RAPD2-PCR. Five distinct genotypes of* P. pentosaceus* were identified in samples originating from the Nemea region. Peza samples harbored a single* P. pentosaceus* genotype, which was also found in Nemea suggesting that it may be a cosmopolitan genotype. The species* Lactobacillus graminis*,* Lactococcus lactis*, and* Weissella* sp. were only detected in the Nemea region. The number of isolates analysed, the distinct banding patterns per population, and the percentage of biodiversity are summarized in Table 2.

Three different bacterial populations were associated with spontaneously fermenting wines in winery tanks. These included 12, 23, and 3 distinct genotypes for* O. oeni*,* P. parvulus*, and* Lactobacillus hilgardii*, respectively. The number of genotypes identified in different tanks is presented in Table 3. One up to five* O. oeni* distinct genotypes were isolated from the same tank. The respective range for* P. parvulus* was 2 to 7. In the case of* Lactobacillus hilgardii* all different genotypes were isolated from the same tank. Present results suggest that the genetic biodiversity of LAB species within a winery may be quite high (Table 3). Most importantly, different strains of the same LAB species may coexist in the same tank during MLF.

### 3.5. Technological Characterization

Distinct genotypes within each species were evaluated as per their technological and enological characteristics (Table 4). Among LAB species, only* O. oeni* and* P. parvulus* isolates were able to grow at low pH, that is, at 3 or 3.5 in the presence of 7% ethanol. Growth at pH 4 was supported by all other species, albeit at different percentages. Winery-associated species showed higher resistance to SO_2_ than vineyard-associated isolates. Among the latter, several isolates of* Lactobacillus plantarum*,* Lactococcus lactis*, and* Pediococcus pentosaceus* tolerated up to 30 mg/L SO_2_.* Lactobacillus graminis* exhibited a moderate resistance, while* Weissella* sp. could grow only up to 30 mg/L SO_2_. Differences between winery- and vineyard-associated species were more profoundly reflected in ethanol tolerance. All vineyard-associated isolates could grow only up to 10% ethanol, except* Weissella* sp. A percentage of 43% of* Lactobacillus plantarum* strains could withstand 12% ethanol. Yet, winery-associated isolates could be considered as highly ethanol tolerant, resisting up to 14% ethanol. Again, the winery-associated isolates showed higher resistance to CuSO_4_ than vineyard-associated isolates did.* P. pentosaceus* was the most sensitive species to CuSO_4_, as none of the strains could tolerate a concentration of 20 mg/L.

### 3.6. BA-Producing LAB

LAB are the main producers of biogenic amines (BAs) in wine. Therefore, LAB should be evaluated for their ability to produce BAs, before being used as malolactic starters. By using appropriate culture media [26], we analysed the different strains identified in this study for their ability to produce the three major BAs in wine, that is, putrescine, tyramine, and histamine. As it is shown in Table 4, except for* Lactobacillus plantarum*,* P. parvulus*, and* Weissella* sp., certain strains from the other species were able to produce putrescine. The percentage of putrescine-producing strains was rather low, except for* Pediococcus pentosaceus*. Tyramine was found to be produced only by* Lactobacillus hilgardii* strains.

Recently, a PCR method was developed for the simultaneous detection of four genes involved in the production of the above BAs [24]. We applied this multiplex PCR to screen the above LAB strains. The PCR results were in good agreement with those obtained by the culture method. There was only one mismatch regarding a* Lactobacillus hilgardii* strain that produced tyramine but the corresponding gene (*tyrdc*) was not amplified. Thus the percentage of mismatching was rather low (1.5% of the strains), being slightly lower than the one detected by Coton et al. [24] (2.5%). It is likely that this discrepancy may be attributed to the existence of novel BA-producing genes not amplifiable by the present degenerate primers [24]. The relatively low frequency of BA-producing strains identified in this study is in accordance to previous results for wine-associated LAB, particularly as regards the low percentage of histamine-producing strains [24].

All three* Lactobacillus hilgardii* strains isolated from one winery tank performing spontaneous MLF produced tyramine and/or putrescine. Present results show the necessity of controlling the MLF by selected starters in order to avoid BA accumulation in the final product, since spontaneous fermentation may allow the occurrence of BA-producing strains.

## 4. Conclusions

The present study shows that the LAB species richness and population densities on grapes may differ considerably between regions or vineyards. Yet,* Lactobacillus plantarum* was the most abundant species in both regions and dominated the alcoholic fermentations. However, there was not any genetic structure in the* Lactobacillus plantarum* populations examined. As expected,* O. oeni* was quantitatively the principal LAB in the winery tanks during the MLF. Present results point to relatively high genotypic and phenotypic diversity within most LAB species identified, including* O. oeni*. Most importantly, various strains of the same species may coexist in the same tank during the MLF. Winery-associated species showed higher resistance to low pH, ethanol, SO_2_, and CuSO_4_ than vineyard-associated isolates. Most LAB strains did not produce BAs in our tests. Further PCR analysis targeting BA-producing genes verified that the frequency of BA-producing LAB was low. However, a few LAB strains isolated from a winery tank conducting MLF did produce major BAs, strengthening the need for novel superior LAB starters to control the MLF.

## Figures and Tables

**Table 1:** Species identification of bacteria isolates based on 16S-ARDRA profiles and sequence analysis.

Profile	Approximate Sizes of Restriction Fragments (bp)	Species
I	610 + 280 + 260 + 190 + 130 + 90	*Lactobacillus graminis *
II	420 + 270 + 200 + 130 + 110 + 90	*Lactobacillus hilgardii *
III	480 + 290 + 270 + 160 + 140 + 110 + 90	Lactobacillus plantarum^*^
IV	400 + 380 + 270 + 180 + 160 + 140	*Lactococcus lactis *
V	610 + 250 + 200 + 130	*Oenococcus oeni *
VI	400 + 270 + 230 + 150 + 130 + 80	*Pediococcus parvulus *
VII	290 + 260 + 250 + 130 + 120 + 110 + 90	*Pediococcus pentosaceus *
VIII	610 + 410 + 290 + 140 + 80	*Staphylococcus epidermidis *
IX	400 + 270 + 240 + 200 + 140 + 80	*Weissella *sp.

^*^ *Lactobacillus plantarum* was differentiated from *L. pentosus *and* L. paraplantarum* with a multiplex PCR assay using *rec*A gene-derived primers.

**Table 2:** Distinct genotypes according to RAPD2-PCR patterns of vineyard-associated LAB populations.

LAB species	Region of origin	No. of isolates	No. of distinct patterns	Percentage of biodiversity^*^	Common patterns among vineyards	Common patterns between regions
*Lactobacillus plantarum *	Nemea Peza	64 319	3 13	4.7 4.1	1 3	2

*Pediococcus pentosaceus *	Nemea Peza	61 16	5 1	8.2 6.3	4 —	1

*Lactobacillus graminis *	Nemea Peza	37 nd^**^	5 —	13.5 —	— —	—

*Lactococcus lactis *	Nemea Peza	21 nd	3 —	14.3 —	— —	—

*Weissella *sp.	Nemea Peza	11 nd	2 —	18.2 —	— —	—

^*^Ratio between the number of patterns and the number of isolates [45].

^**^Not detected.

**Table 3:** Distinct genotypes according to RAPD2-PCR patterns of winery-associated LAB populations.

LAB species	Tank (T1–T9)	No. of isolates	No. of distinct patterns	Percentage of biodiversity^*^
*Lactobacillus hilgardii *	T6	4	3	75.0
*Oenococcus oeni *	T1–T8	46	12	26.1
*Pediococcus parvulus *	T1–T6	38	23	60.5

^*^Ratio between the number of patterns and the number of isolates [45].

**Table 4:** Technological characteristics and biogenic amines production of vineyard- and winery-associated LAB species. The total number of strains analysed per species and the number of strains that produced positive reactions are indicated.

LAB species	No of strains	Biogenic amines			pH			SO_2_ (mg/L)			Ethanol (%)			CuSO_4_ (mg/L)	
		Putrescine	Tyramine	Histamine	3.0	3.5	4	5	15	30	10	12	14	5	20
*Lactobacillus graminis *	5	1	0	1	0	0	2	2	2	2	1	0	0	2	1
*Lactobacillus hilgardii *	3	1	3	0	0	0	3	3	3	3	3	3	2	3	3
*Lactobacillus plantarum *	14	0	0	0	0	0	11	11	11	8	8	6	0	11	4
*Lactococcus lactis *	3	1	0	0	0	0	3	3	3	3	1	0	0	3	3
*Pediococcus parvulus *	23	0	0	0	17	21	22	21	21	21	21	21	21	21	21
*Pediococcus pentosaceus *	5	5	0	0	0	0	5	5	5	2	4	0	0	5	0
*Oenococcus oeni *	12	0	0	0	3	9	11	10	10	6	12	10	10	10	9
*Weissella *sp.	2	0	0	0	0	0	1	2	1	0	0	0	0	1	1
